# Supplementary material for: Abrupt increase in Arctic-Subarctic wildfires caused by future permafrost thaw
Source: Nat Commun. 2024 Sep 24;15:7868. doi: 10.1038/s41467-024-51471-x (PMC11422492; doi:10.1038/s41467-024-51471-x)
Supplement: Supplementary file 1 — Supplementary Information [file 41467_2024_51471_MOESM1_ESM.docx]

Supporting Information for

**Abrupt increase in Arctic-Subarctic wildfires caused by future permafrost thaw**

In-Won Kim^1,2*^, Axel Timmermann^1,2^, Ji-Eun Kim^1,2^, Keith B. Rodgers^3^, Sun-Seon Lee^1,2^, Hanna Lee^4^, and William R. Wieder^5,6^

1 Center for Climate Physics, Institute for Basic Science, Busan, Republic of Korea, 46241

2 Pusan National University, Busan, Republic of Korea, 46241

3 WPI-Advanced Institute for Marine Ecosystem Change, Tohoku University, Sendai, Japan

4 Norwegian University of Science and Technology, Trondheim, Norway

5 Climate and Global Dynamics Laboratory, National Center for Atmospheric Research, Boulder, CO, USA 80307

6 Institute of Arctic and Alpine Research, University of Colorado Boulder, Boulder, CO, USA 80309

Corresponding author: In-Won Kim ([iwkimi@pusan.ac.kr)](mailto:email@address.edu))


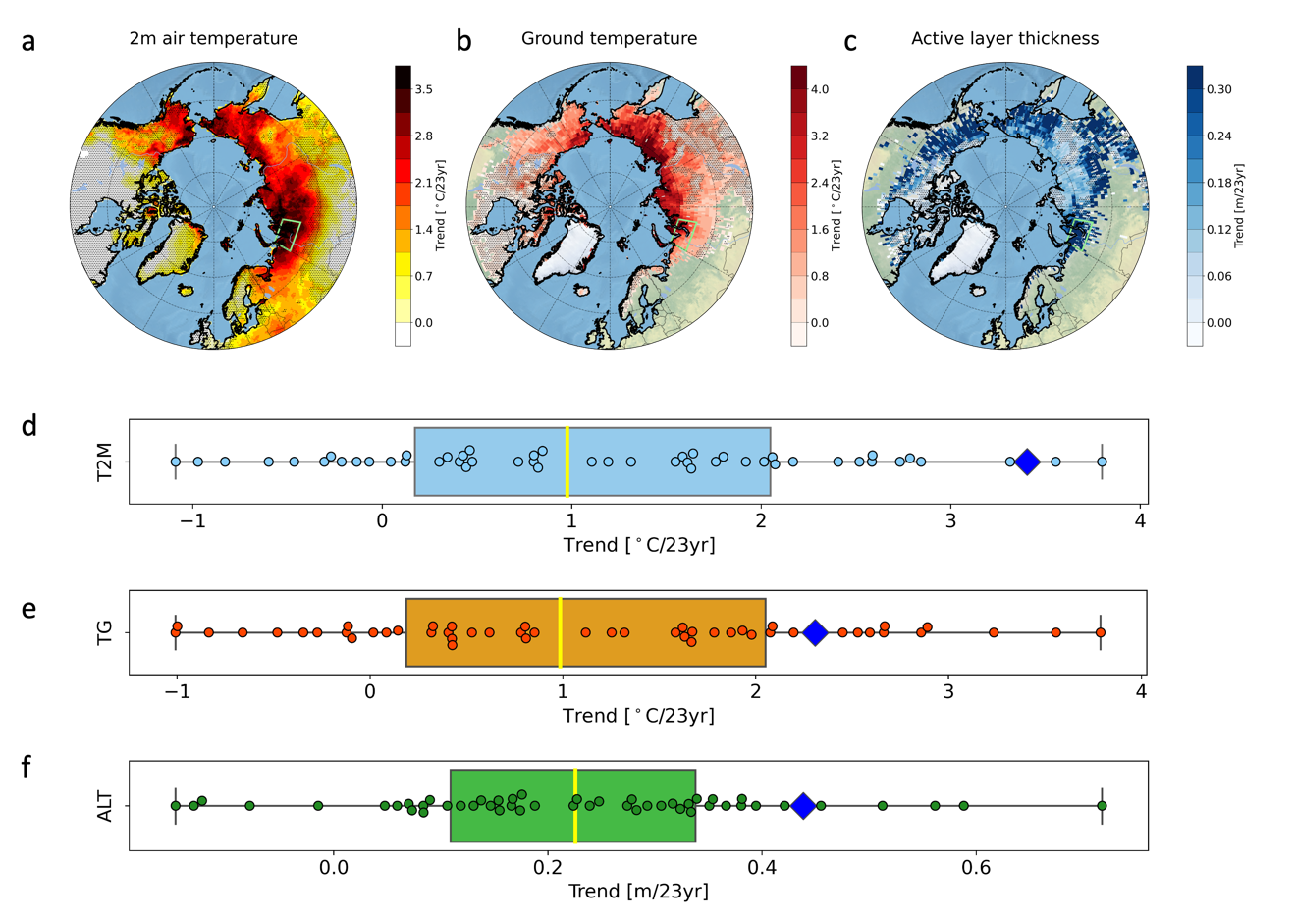


**Supplementary Figure 1. Trends of 2m air temperature (T2M), ground temperature (TG), and active layer thickness (ALT) over the period 1997-2019 in reanalysis datasets over the historical permafrost regions.** Trends in (a) the ERA5 T2M (units: °C/23year), (b) the CCI-PF TG (units: °C/23year), and (c) the CCI-PF ALT (units: m/23year). Stippling indicates areas where the linear trend is statistically significant at a 5% significance level. Boxplot of trends in the 50 ensemble members of the CESM2-LE over Western Siberia (65-70°N, 60-80°E, marked by the light-green box outline in the map): (d) T2M (units: °C/23year), (e) TG (units: °C/23year), and (f) ALT (units: m/23year). Blue square markers indicate trends in the reanalysis datasets and circle markers indicate trends in the 50 individual ensemble members. Colored boxes represent 25–75% quartiles. Medians are shown as yellow solid lines.

**
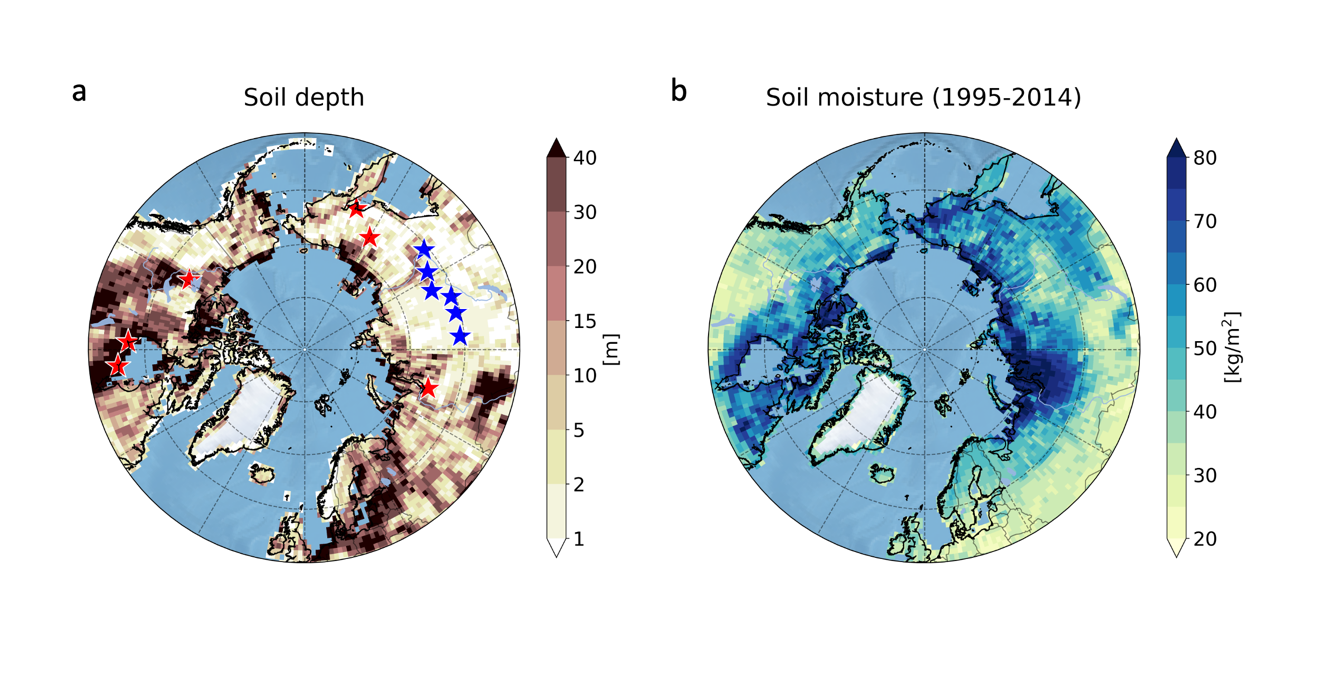
 Supplementary Figure 2. Arctic-Subarctic soil properties.** (a) Soil depth in the CESM2-LE (unit: m) and (b) climatological mean of soil moisture over 0-10cm depth (unit: kg/m^2^) for 1995-2014 in the CESM2-LE. Red star markers in the panel indicate the regions with deep soils (65.5°N, 72.5°E; 65.5°N, 150.0°E; 61.73°N, 165.0°E; 55.13°N, 85.0°W; 57.02°N, 92.5°W; 64.55°N, 121.2°W). Blue star markers in the panel indicate the regions with shallow soils (60.79°N, 95.0°E; 60.79°N, 103.8°E; 60.79°N, 110.0°E; 63.61°N, 115.0°E; 62.67°N, 122.5°E; 60.79°N, 130.0°E).

**
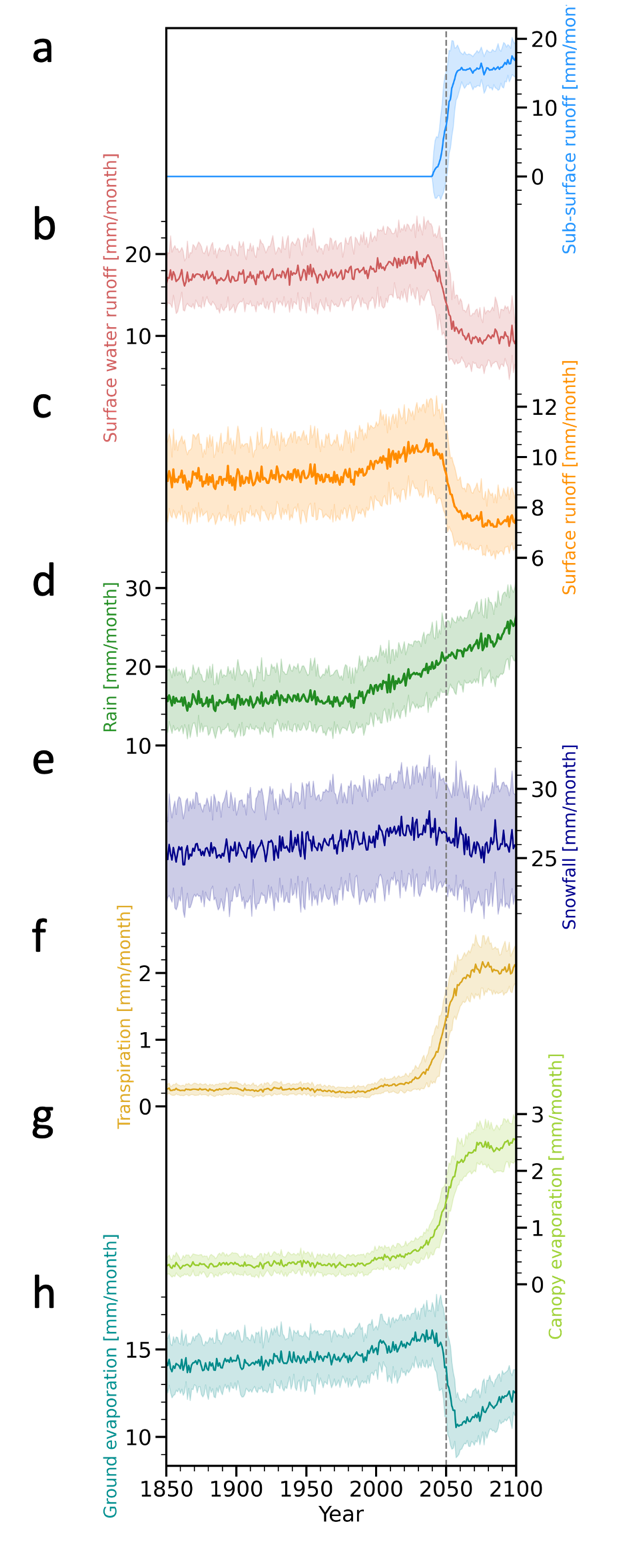
**

**Supplementary Figure 3. Time evolution of mean annual hydrological components in Western Siberia (65.5°N, 83.75°E) among 50 ensemble members.** (a) Sub-surface runoff (units: mm/month), (b) surface water storage runoff (units: mm/month), (c) surface runoff (units: mm/month), (d) rainfall (units: mm/month), (e) snowfall (units: mm/month), (f) canopy transpiration (units: mm/month), (g) canopy evaporation (units: mm/month), and (h) ground evaporation (units: mm/month). Bold lines indicate ensemble means and shading indicates ±1 standard deviation of 50 ensemble members.


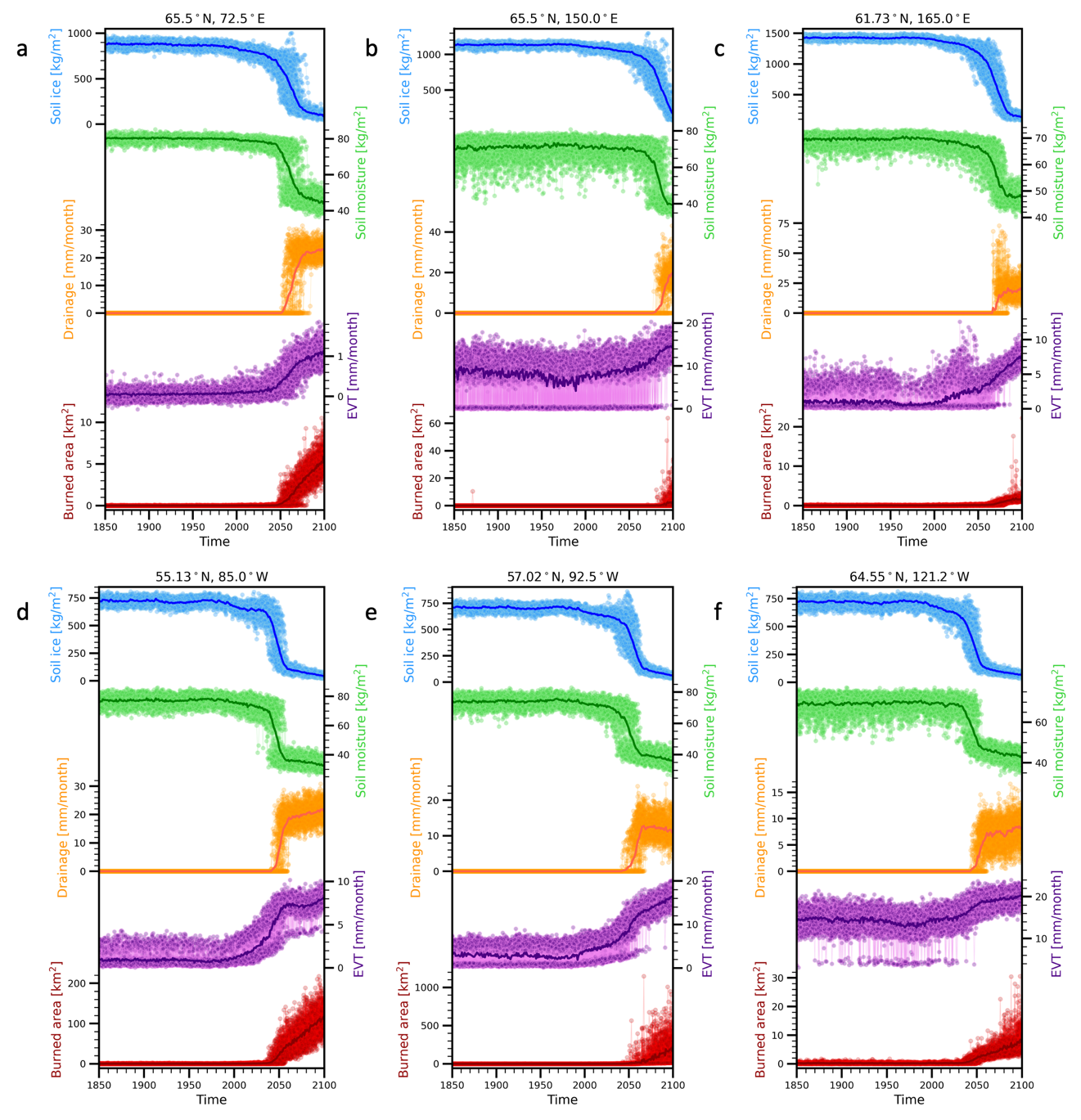
 **Supplementary Figure 4. Time evolution of rapid changes over the permafrost regions among 50 ensemble members. (**a) 65.5°N, 72.5°E, (b) 65.5°N, 150.0°E, (c) 61.73°N, 165.0°E, (d) 55.13°N, 85.0°W, (e) 57.02°N, 92.5°W, and (f) 64.55°N, 121.2°W (Red star markers in Fig.S2a), blue: soil ice content (units: kg/m^2^), green: soil moisture in 0-10cm depth (units: kg/m^2^), yellow: sub-surface runoff (units: mm/month), purple: canopy evapotranspiration (units: mm/month), red: burned area (units: km^2^). Bold lines indicate the ensemble mean and thin lines indicate individual 50 ensemble members.


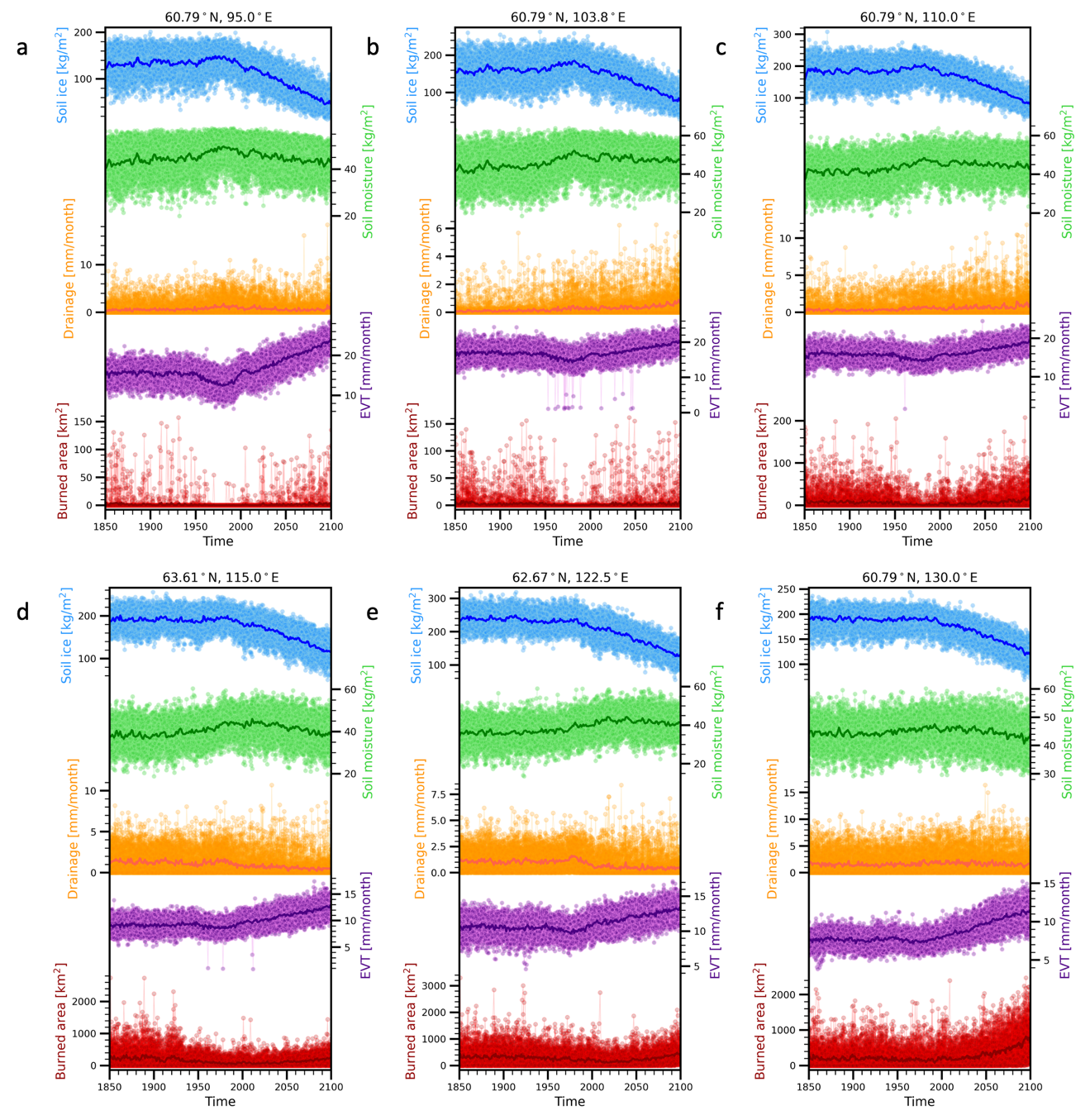
 **Supplementary Figure 5. Time evolution of gradual changes over the permafrost regions among 50 ensemble members.** (a) 60.79°N, 95.0°E, (b) 60.79°N, 103.8°E, (c) 60.79°N, 110.0°E, (d) 63.61°N, 115.0°E, (e) 62.67°N, 122.5°E, and (f) 60.79°N, 130.0°E (Blue star markers in Fig.S2a), blue: soil ice content (units: kg/m^2^), green: soil moisture in 0-10cm depth (units: kg/m^2^), yellow: sub-surface runoff (units: mm/month), purple: canopy evapotranspiration (units: mm/month), Red: burned area (units: km^2^), Bold lines indicate ensemble mean and thin lines indicate individual 50 ensemble members.

**Supplementary Figure 6. Time evolution in Western Siberia (65.5°N, 83.75°E) in July.** (a) Ground heat flux into the soil layers (units: W/m^2^), (b) surface air temperature (units: °C), and (c) specific humidity at 2m (units: kg/kg). Bold lines indicate ensemble means and shading indicates ±1 standard deviation across 50 ensemble members.

**
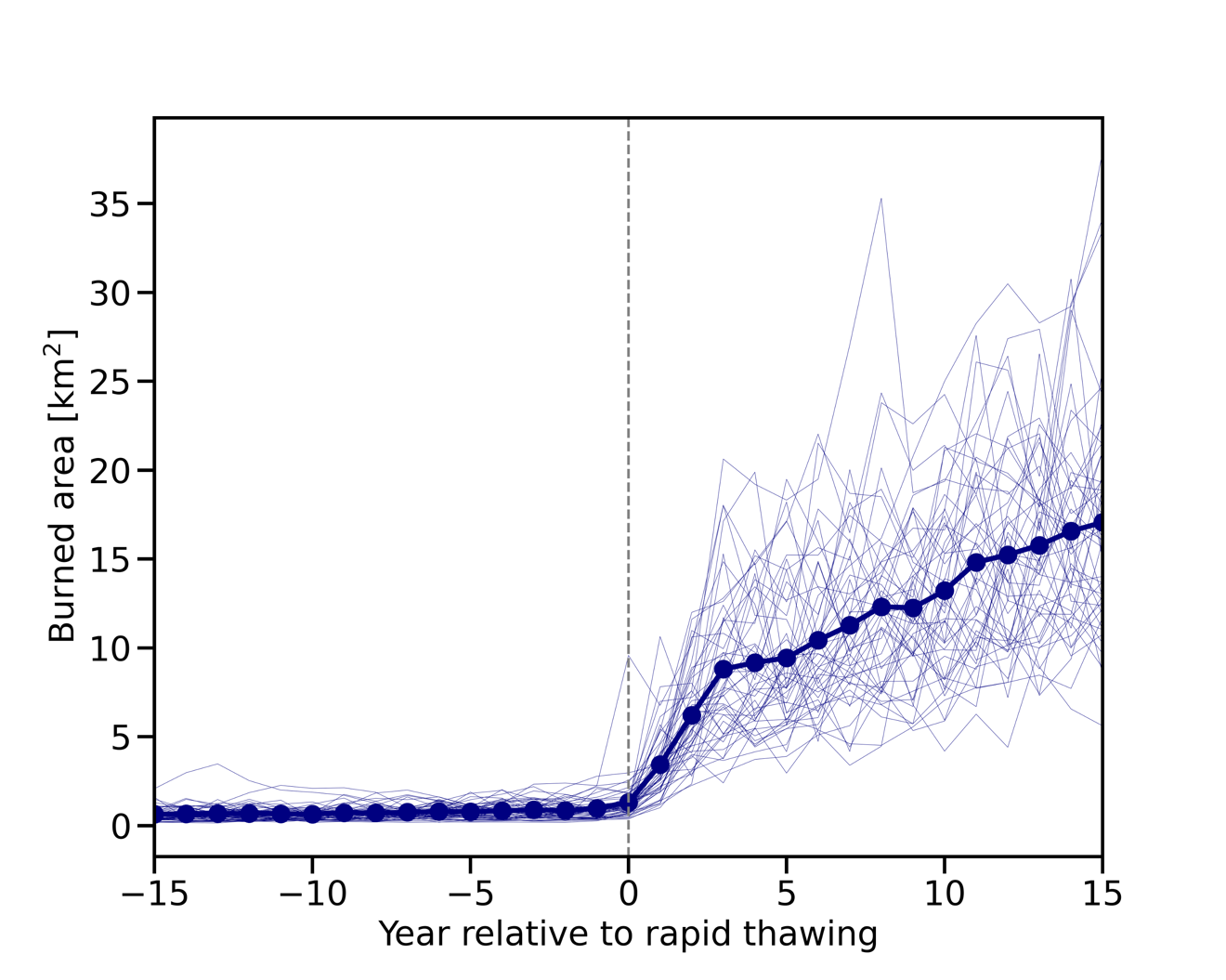
**

**Supplementary Figure 7. Changes in the burned area during the period of rapid permafrost thaw within the historical permafrost regions (time interval 2020 to 2085).** The zero point on the x-axis represents the timing of the occurrence of rapid permafrost thaw and the y-axis indicates the burned area (units: km^2^). The bold line indicates the ensemble mean and the thin lines indicate 50 ensemble members.


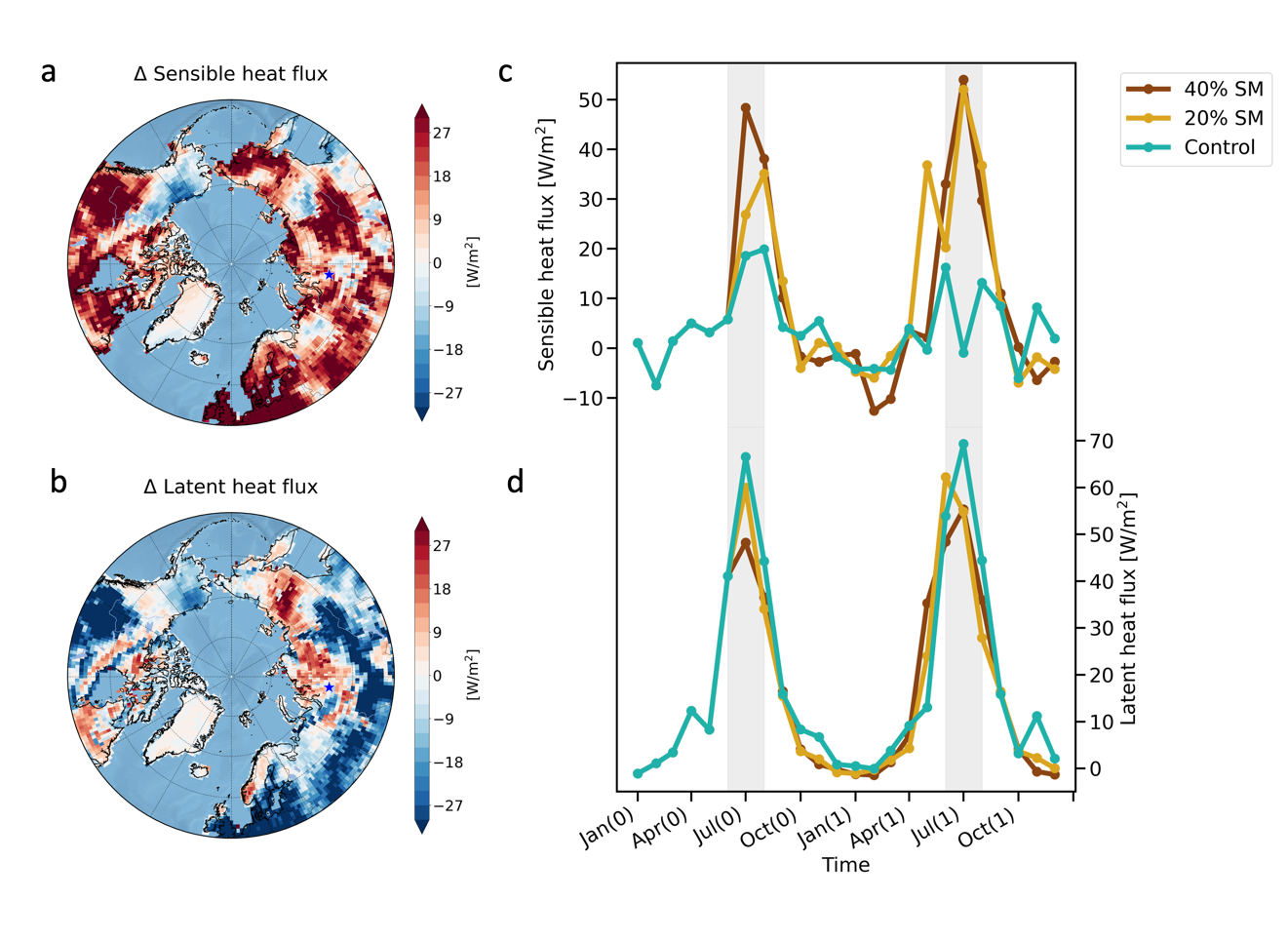
**Supplementary Figure 8. Turbulent heat fluxes responses to soil moisture reduction in the idealized experiments with the CESM2.** Values represent differences between the response of the 40% soil moisture reduction perturbation experiment in 2045 and a control simulation (a-b): (a) sensible heat flux (units: W/m^2^), (b) latent heat flux (units: W/m^2^). Time evolution over Western Siberia (65.5°N, 83.75°E) (c-d): (c) sensible heat flux (units: W/m^2^), and (d) latent heat flux (units: W/m^2^), blue: control simulation, yellow: 20% soil moisture reduction perturbation experiment, brown: 40% soil moisture reduction perturbation experiment.

**
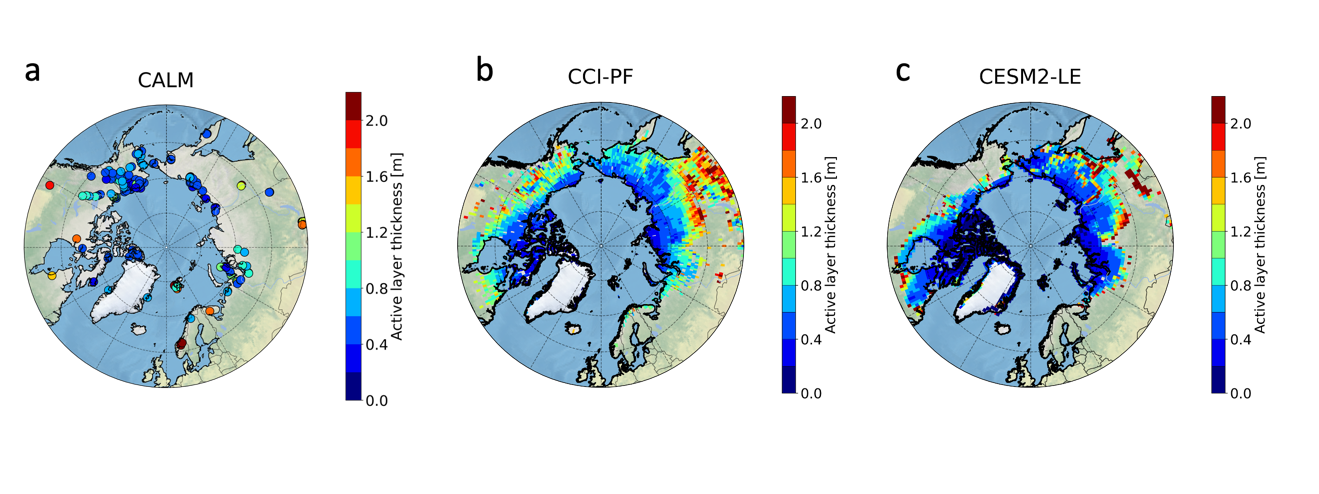
**

**Supplementary Figure 9. Comparison of active layer thickness (ALT) between observations and the CESM2-LE simulations for the period of 1997-2014.** The climatological mean of ALT from (a) the CALM sites, (b) the CCI-PF, and (c) the CESM2-LE (units: m).

**
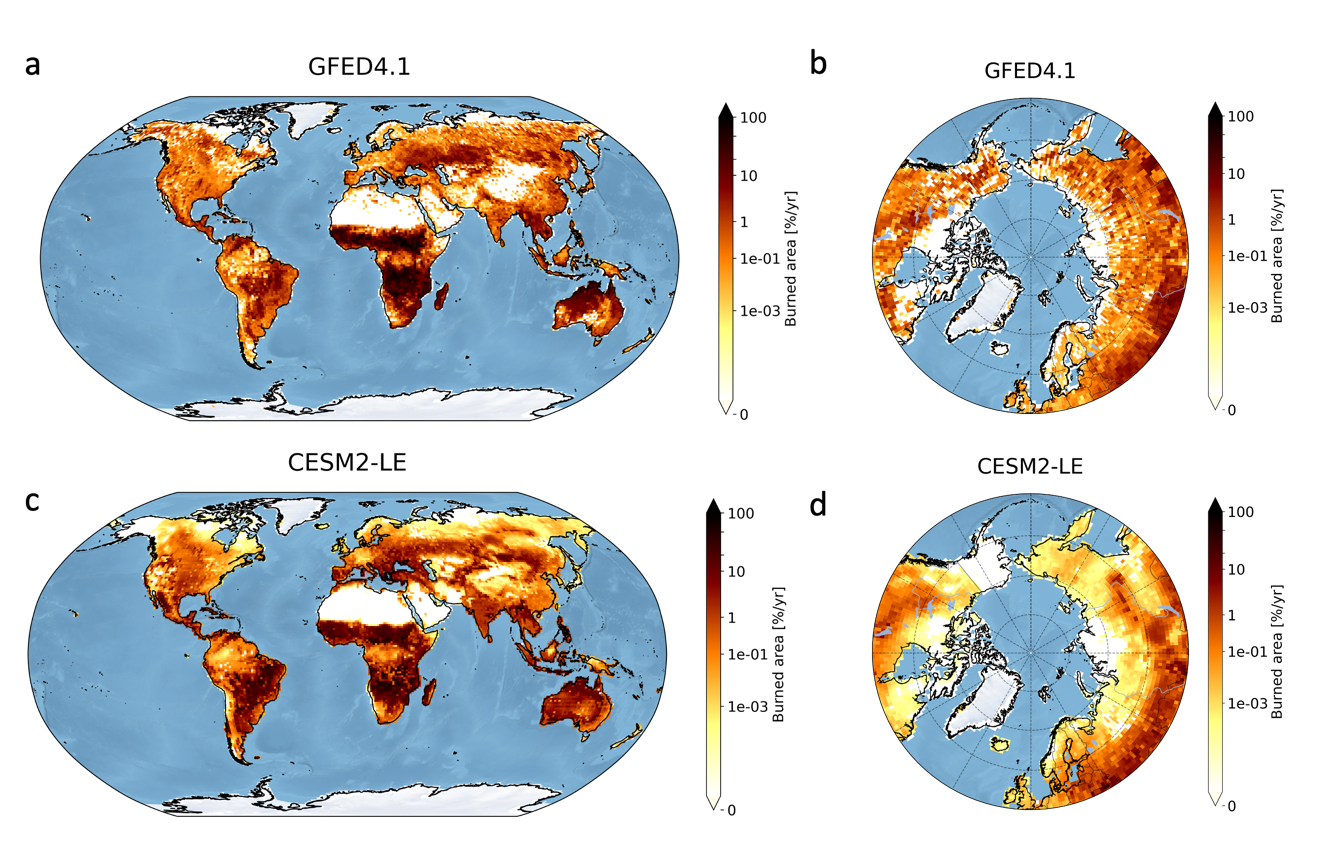
**

**Supplementary Figure 10. Comparison of burned area between observation and the CESM2-LE simulations averaged over the period 1997-2014.** The climatological mean of the burned area from (a, b) the GFEDv4.1 and (c, d) the CESM2-LE (units: %/year).
